# Psychological trauma and help seeking behaviour amongst resettled Iraqi refugees in attending English tuition classes in Australia

**DOI:** 10.1186/1752-4458-9-5

**Published:** 2015-01-20

**Authors:** Shameran Slewa-Younan, Jonathan M Mond, Elise Bussion, Maral Melkonian, Yaser Mohammad, Hanan Dover, Mitchell Smith, Diana Milosevic, Anthony Francis Jorm

**Affiliations:** Mental Health, School of Medicine, The University of Western Sydney, Locked Bag 1797 Penrith South DC, NSW Australia; Department of Psychology, Macquarie University, Sydney, Australia; Mental Health, School of Medicine, The University of Western Sydney, Sydney, Australia; New South Wales Refugee Health Service, Liverpool, UK; South Western Sydney Local Health District Eastern Campus, Liverpool Hospital, Sydney, Australia; Centre for Mental Health, Melbourne School of Population and Global Health, University of Melbourne, Melbourne, Australia

**Keywords:** Refugees, Trauma, Posttraumatic stress disorder, Psychological distress, Mental health, Resettlement

## Abstract

**Background:**

To examine levels of psychological distress and help seeking behaviour in resettled refugees attending English tuition classes in Australia, and their associations with participants’ demographic characteristics.

**Methods:**

Data was collected by bilingual interviewers between March and November 2013. A volunteer sample of attendees of Adult Migrant English Programs (AMEP) in Western Sydney were recruited. Participants were two hundred and twenty five Iraqi refugees resettled in Western Sydney, who had left Iraq no earlier than 1991, were fluent in Arabic and/or English, and were between the ages of 18 and 70. The chief outcome measures used were the Kessler Psychological Distress Scale (K-10) as well as The Harvard Trauma Questionnaire (HTQ).

**Results:**

On the K-10, 39.8% of participants had severe psychological distress, 19.4% moderate distress, and 40.7% had low to mild distress. Ninety-five percent of participants reported having experienced one or more potentially traumatic event (PTE) as defined by the HTQ prior to leaving Iraq, with a mean of 14.28 events (SD = 8.69). Thirty-one percent of participants met the threshold (≥2.5) for clinically significant PTSD symptomatology, with a significantly higher occurrence among participants with lower education attainment (*χ*^*2*^ (3) = 8.26, *p* = .04). Of those participants with clinically significant PTSD symptomatology according to the HTQ, only 32.9% reported ever having ever sought help for a mental health problem.

**Conclusions:**

The high level of distress found in this sample, combined with low uptake of mental health care, highlights the need for programs targeted to promote help-seeking among Iraqi refugees who have resettled in Australia. Further, the higher level of PTSD symptomatology found amongst those with lower education attainment has mental health promotion and treatment implications. Specifically, in designing service and treatment programs, consideration should be given to the possible impact excessive levels of psychological distress may have on learning in refugees, to ensure that those who have been unable to develop proficiency in the English language receive effective care.

## Background

Resettled refugees are a particularly vulnerable group in society. Each year Australia offers protection to approximately 13,500 refugees and to date Australia has resettled 700,000 since the end of World War II [1]. A refugee is a person who has left their home country as a result of *“a well founded fear of persecution for reasons of race, religion, nationality, membership of a particular social group or political opinion”*[2]. This persecution often involves serious human rights abuses, including torture, imprisonment and violence.

Psychological distress, in particular Posttraumatic Stress Disorder (PTSD) and symptoms of depression, is known to be common in refugee populations [3–5]. In a meta-analysis of 181 studies of refugee and conflict-affected populations, Steel and colleagues [5] reported that the weighted prevalence of PTSD and depression were, respectively, 30.6% and 30.8%. Factors associated with variation in prevalence included torture, cumulative exposure to potentially traumatic events (PTEs), time since conflict and the level of political terror in country of origin.

After resettlement, the physical and psychological health of refugees presents a new area of concern. Refugees experience a complex set of mental health issues owing to their pre-migration experiences, as well as post-migration cultural dislocation [5]. These mental health concerns require a targeted response from the local community and the health professionals that service it. However, there is evidence to suggest a lack of equity with respect to access to health services [6]. In a recent study comparing annual, age-standardised hospital admission rates in Victoria for people born in refugee-source countries and Australian-born people, Correa-Velez and colleagues [7, 8] found that participants from a refugee background were 30% less likely to have mental or behavioural admissions than those born in Australia. This is consistent with data on service utilisation in other nations, where similar disparities in health service utilisation were reported [9–12].

One group of potentially severely traumatized refugees are those who originate from Iraq. Iraq has been Australia’s primary refugee country of origin since 2008, with 14,378 Iraqi refugees resettled in Australia since this time [13]. The ongoing Iraqi conflict has displaced 1 in 7 Iraqis, totalling 4.2 million, a displacement that represents one of the largest refugee populations ever processed by the United Nations High Commissioner for Refugees (UNHCR) [14]. The past ten years of conflict have left many Iraqi refugees experiencing the after-effects of pre-migration trauma such as targeted violence and torture [15]. In a study of Mandean Iraqi refugees resettled in Australia, participants had experienced on average 4.3 different forms of trauma according to the Harvard Trauma Questionnaire (HTQ). Frequently reported traumatic experiences included the murder of a family member or friend (39%), deprivation of food and water (42.8%), and being close to death (49.5%) [16]. Although, there was no assessment of help-seeking behaviour in this study, mental health care appeared to be uncommon. In the study of Correa-Velez and colleagues, referred to above, psychiatric admissions among Iraq-born patients were lower than those for all other refugee groups over a 6-year period [8].

As part of the resettlement process, host nations often provide a variety of services including language tuition for those with low proficiency in the host language. In Australia, Adult Migrant English Program (AMEP) classes are delivered by service providers around Australia in over 250 locations. The program involves attending 510 h of English language training, career counselling and task-based learning aimed at facilitating living and settlement in Australia [17]. Although, no specific data has been published on the psychological health of attendees of the AMEP program, previous research has found that levels of psychological distress impede the ability to learn language in refugee populations [18, 19]. It is postulated that this may be due to the simultaneous conflicting demands on working memory capacity, particularly between language retention and distressing cognitive operations [20]. Following on from research demonstrating that early recognition and intervention is optimal in the treatment of mental health conditions [21], we suggest that consideration of levels of psychological distress demonstrated by resettled refugees attending language tuition classes is important in allowing the development of better mental health systems that can address and aid refugee resettlement.

Given that Iraqi continue to constitute a significant proportion of those seeking asylum, in light of the continuing conflict within Iraq, information about this population’s mental health status and help-seeking behaviour is important for health professionals and for public health administrators. Consequently, the goal of this study was to examine levels of general psychological distress, including PTSD symptomatology, and help seeking behaviour in a volunteer sample of Iraqi refugees attending English tuition classes in Australia. We were also interested to consider associations between mental health and help-seeking and individuals’ demographic characteristics, since associations of this kind might indicate specific targets for health promotion and/or early intervention programs [22].

## Methods

### Study design and participants

Participants were resettled Iraqi refugees attending the Adult Migrant English Program (AMEP) at three different colleges located in the cities of Auburn, Fairfield and Liverpool which are in the Western Sydney region of Australia. AMEP is a federally funded English language tuition program that is offered to all migrants and humanitarian entrants to Australia who do not have functional English. Participation entailed the completion of a pen-and-paper, respondent-based survey, conducted on the college campuses by means of in-person interviews. Bilingual investigators attended the colleges between March and November 2013 and informed potential participants of the project through the distribution of flyers. The flyers outlined the aims and nature of the research and indicated that all participants would receive a food gift voucher in the amount of AUD $25.00 upon completion of the survey in appreciation of their time. Written consent was obtained from all participants, following a more detailed description of the survey content, prior to commencement of each interview. Inclusion criteria were having been born in Iraq, having left Iraq no earlier than 1991, being fluent in Arabic and/or English, and being between the ages of 18 and 70 years. Approval for the study was obtained from the University of Western Sydney Human Research Ethics Committee.

The survey included, in addition to demographic information and measures of mental health as described below, a “mental health literacy” component in which a vignette was presented of a fictional Iraqi refugee, who had been exposed to trauma prior to leaving Iraq and who was suffering symptoms of PTSD, followed by a series of questions concerning the problem described [22]. One of these questions asked whether participants had ever sought help for a problem such as the one described and, if so, from which person(s) or treatment services they had sought help. Interviews were conducted by two members of the research team fluent in Arabic and English and extended up to 90 min.

### Measures

#### General psychological distress

To assess the general symptoms of anxiety and depression, the Kessler Psychological Distress Scale (K10) [23] was used. The K10 is a self-reported questionnaire of depression and general mental disorder. Scores range from 10 to 50, with established thresholds of low to mild (10–21), moderate (22–29) and severe distress (≥30) applied to provide a measure of symptoms among the participants. The K10 has good psychometric properties with internal consistency of α = .86 reported for Arabic speaking refugees [24]. Cronbach’s alpha in the current study was .94.

#### Trauma and PTSD symptoms

The Harvard Trauma Questionnaire (HTQ) [25] is a widely used, cross-cultural self-report checklist assessing the occurrence and frequency of various types of traumatic events as well as PTSD symptomatology. It consists of four parts, however only parts I (assessing the number and types of PTEs experienced and/or witnessed) and IV (addressing the occurrence and severity of PTSD symptomatology) were included in the present study. A score of 2.5 or above on Part IV is considered to indicate a high probability of clinically significant PTSD symptomatology [24]. The HTQ has very good psychometric properties, including high test-retest reliability and internal consistency for part IV [25] and has been validated for Iraqi community [26]. Cronbach’s alpha in the current study was .93.

#### Statistical analyses

Statistical analysis was carried out using IBM SPSS Statistics version 20.0 [27]. Continuous variables are presented as means ± standard deviation (SD), whereas categorical variables were expressed as percentage (%) frequencies. Subgroups were examined using independent samples t-tests, ANOVA, Mann–Whitney U tests, Kruskal-Wallis tests, or Chi-square tests, as appropriate. Missing data was low, in the order of 7.2% and was handled by listwise deletion in the association analyses. Where appropriate, data for symptoms of general psychological distress and PTSD symptomatology derived from the second (2007) Australian National Survey of Mental Health and Wellbeing [28] were used for comparative purposes.

## Results

Interviews were conducted with a total of 225 participants over a 10-month data collection period. Their demographic characteristics are shown in Table 1.

**Table 1:** **Demographic characteristics of participants** (**N** = **225**)

Demographic Characteristics	N (%)
Sex	
Male	98 (43.6%)
Female	127 (56.4%)
Age in years (Mean, SD)	37.88 (14.16)
Years of Education (Mean, SD)	10.68 (3.90)
Months in Australia (Mean, SD)	59.11 (64.54)
Months externally displaced (Mean, SD)	49.86 (70.42)
Religion	
Christian	102 (45.1%)
Muslim	86 (38.1%)
Mandean	37 (16.4%)
Marital Status	
Never married	51 (22.6%)
Married/Partner	151 (66.8%)
Divorced	5 (2.2%)
Widowed	12 (5.3%)
Not indicated	5 (2.2%)

### K10 results

According to the cut-off scores for the K10 used in the Australian National Survey of Mental Health and Well-Being (NSMHWB) [28] 39.8 % of participants had severe psychological distress, 19.4% had moderate distress, 40.7 % had low to mild distress. By way of comparison, 2.6 % of the general Australian population experienced severe psychological distress according to the second NSMHWB. There was a significant association between distress levels and education *χ*^*2*^ (6) = 16.01, *p* = .01. As shown in Table 2, significantly more participants with 6 or less years of education than expected reported experiencing severe distress (*z* = 2.4). There was no association between distress levels and age *χ*^*2*^ (4) = 1.54, *p* = .82, sex *χ*^*2*^ (4) = 2.08, *p* = .72, religion *χ*^*2*^ (4) = 4.83, *p* = .31, or marital status *χ*^*2*^ (6) = 6.12, *p* = .41.

**Table 2:** **Levels of general psychological distress**, **as measured by the Kessler Psychological Distress Scale** (**K**-**10**) **and symptoms of Post-Traumatic Stress Disorder, as measured by the Harvard Trauma Questionnaire (HTQ), among study participants (N = 225) as a function of education**

		K-10		HTQ	
	N ^i^	Mean (SD)	High distress ^ii^ (%)	Mean (SD)	Probable PTSD ^iii^ (%)
Years of education:					
6 or less	36	30.63(12.13)	62.2	2.35(0.76)	47.5
7-11	75	25.64(10.64)	41.3	1.91(0.77)	26.9
12-15	72	23.71(9.57)	28.2	1.94(0.69)	24.0
16 or more	29	23.97(10.06)	31.0	2.14(0.62)	34.5

^i^Does not equal to 225 due to missing data.

^ii^K-10 ≥ 30.0.

^iii^HTQ ≥ 2.5.

### Total potentially traumatic events (PTE’s) experienced

Ninety-five per cent of participants reported having experienced one or more PTE’s prior to leaving Iraq (*M* = 14.28, *SD* = 8.69). The most commonly reported events were being forced to flee country (81.9 %), being confined to home because of chaos and violence outside (77.9 %), and being oppressed because of ethnicity, religion or sect (69.9 %). The total number of PTEs experienced did not differ significantly by sex, t (222) = 0.75, p = .45), education, F (3,218) = 1.88, *p* = .13, religion, H (2) = 3.46, *p* = .18, or marital status, F (5,218) = 0.51, *p* = .51. Total PTEs did, however, vary as a function of age, in that older participants (those older than 50 years) experienced more PTEs, F (2, 217) = 13.98, *p* < .001.

### PTSD symptoms

Thirty-one per cent of participants met the threshold (≥2.5) for clinically significant PTSD symptomatology according to the HTQ. In comparison, reported rates of PTSD in NSMHW ranged from 6.4 % for 12 months prevalence to 12.2% for lifetime presence, which are between three to five fold less than our sample. There was a significant association between PTSD symptoms and education *χ*^*2*^ (3) = 8.26, *p* = .04. As noted in Table 2, significantly more participants with 6 or less years of education than expected reported experiencing probable PTSD (*z* = 2.0). There was no association between PTSD symptoms and age, *χ*^*2*^ (2) = .698, *p* = .71, sex *χ*^*2*^ (2) = 0.52, *p* = .47, religion *χ*^*2*^ (2) = 0.34, *p* = .85, or marital status *χ*^*2*^ (3) = 7.88, *p* = 0.96.

### Help seeking behaviour

Nineteen percent of participants reported having ever sought help for a mental health problem, such as the one described in the vignette. Of these, the top four sources for seeking help were family member (27.2 %), psychiatrist (16.1%), GP (14.4 %) and psychologist (13.8%). It is interesting to note that 6.5% of participants sought help from their religious leader or clergyman. There was no association between help-seeking behaviour and age *χ*^*2*^ (2) = 2.80, *p* = .25, sex *χ*^*2*^ (2) = 0.45, *p* = .80, religion *χ*^*2*^ (2) = 1.84, *p* = .40, education *χ*^*2*^ (3) = 1.27, *p* = .74, marital status *χ*^*2*^ (3) = 6.75, *p* = .08, or distress level *χ*^*2*^ (2) = 2.61, *p* = .27. However, there was a significant association between help-seeking behaviour and PTSD symptomology *χ*^*2*^ (1) = 11.26, *p* < .01. Significantly more participants with probable PTSD than expected sought help for a mental health problem (*z* = 2.5). Nevertheless, only 32.9 % of participants experiencing PTSD symptomology according to the HTQ reported having ever sought help for a mental health problem. Of those with probable PTSD, the most common type of help sought was from a family member (23.1%), followed by GP (21.5%), psychiatrist (13.8%), psychologist (12.3%) and religious leader (10.8%). It is interesting to note that only 9.2% of participants reported seeking help from specialist trauma and torture mental health services.

## Discussion

This study sought to report on trauma exposure and associated psychological symptomatology and help seeking in Iraqi refugees who were attending English tuition classes. This refugee population represents one of the largest groups of refugees currently being resettled in Australia and hence their psychological profile is important from a service provision perspective. Our results indicated that more than 90% of participants reported exposure to at least one human rights violation, 39% reported severe psychological distress and 31% had clinically significant symptoms of PTSD. Less than one in four participants (19.5%) reported ever having sought help for a trauma related mental health problem and, of those participants who had sought such help, only a small minority had consulted a mental health professional (13.8%). Such significantly high levels of psychological distress and trauma exposure amongst the participants bears direct relevance in their ability to effectively engage with and participate in the English tuition classes.

Although the levels of distress and exposure to trauma observed among participants in the current study are comparable to those observed in other resettled refugee populations [5, 29], their reported low uptake of mental health care is concerning. While help-seeking was more common among participants with clinically significant symptoms of general psychological distress and/or PTSD symptomatology, still less than one third of participants in these subgroups had ever sought help in relation to a trauma-related mental health problem. These findings indicate the need for programs designed to promote early, appropriate help-seeking among Iraqi refugees who have resettled in Australia. Given that poor “mental health literacy” is a known barrier to the uptake of evidence-based treatment among individuals with mental health problems [21], research addressing specific aspects of mental health literacy likely to be conducive to low or inappropriate help-seeking in resettled refugee populations would be of interest and we hope to report findings from the current research addressing this issue in due course.

A secondary aim of the current study was to examine associations between distress and PTSD symptomatology and demographic variables and thereby identify subgroups of the refugee population likely to be most vulnerable. Results of this analysis indicated participants with little or no formal education had particularly high levels of distress and traumatic symptomology. This potentially bears consideration in the development of refugee specific service provision and mental health treatment. Firstly, aside from specialist torture and trauma services, many of those who are involved in the resettlement process in Australia (AMEP teachers, community workers) may have a brief understanding of the refugee process but limited understating of the resultant psychological conditions. As such, these frontline workers are poorly equipped to deal with the psychological distress and emergent mental health needs. We propose that additional training and resources, such as a culturally adapted Mental Health First Aid program, be provided to those who work with refugees thereby promoting early identification and first aid in a timely and culturally appropriate manner. A second consideration bears direct relevance to the type of psychological treatment offered to traumatized refugees. Although trauma-focused cognitive behavioural therapy may usually be considered the most effective treatment for PTSD in most traumatized populations [30], its effectiveness relies on engagement and compliance with homework tasks often involving a written component. This may reduce the effectiveness of CBT in those most in need of treatment, due to known limitations to learning resulting from high levels of psychological distress in refugee populations [18–20]. Refugees who have a reduced capacity for acquiring literacy require treatment that caters specifically to their circumstances, with consideration of language as a likely barrier. As such, alternative modalities that do not require literacy on the part of the patient, such as narrative exposure therapy may be preferable for traumatized individuals with very low levels of literacy [31].

Limitations of the current study should be noted. These include the use of self-report measures of mental health, the inclusion of a measure of general psychological distress rather than separate measures of anxiety and affective disorders, and the collection of only basic information concerning help-seeking behaviour. Strengths of the study, in our view, were the relatively large sample size, which permitted analysis of associations between demographic characteristics and measures of mental health and the administration of the survey instrument by means of in-person interviews. Although participants were volunteers attending the AMEP classes, by recruiting from colleges in western Sydney which has the highest concentration of resettling Iraqi refugees in Australia, an attempt at redress representativeness was made.

## Conclusions

In sum, Iraqi refugees attending English classes are likely to have very high levels of distress and PTSD symptomatology and very low uptake of mental health care even among severely distressed individuals. Those with minimal education may be a particularly vulnerable subgroup. There is a need for research to identify aspects of mental health literacy likely to be conducive to low or inappropriate help-seeking in resettled refugee populations and to use this information to inform culturally sensitive health promotion and early intervention programs.

## Abbreviations

AMEP: Adult migrant english programs
K-10: Kessler psychological distress scale
HTQ: The Harvard trauma questionnaire
NSMHWB: Australian national survey of mental health and well-being
PTE: Potentially traumatic event
PTSD: Posttraumatic stress disorder
SD: Standard deviation
UNHCR: United Nations High Commissioner for refugees.
